# Abortions in Germany – Current data from the statistics on terminations of pregnancy

**DOI:** 10.25646/9956

**Published:** 2022-06-29

**Authors:** Franziska Prütz, Birte Hintzpeter, Laura Krause

**Affiliations:** Robert Koch Institute, Berlin, Department of Epidemiology and Health Monitoring

**Keywords:** ABORTION, WOMEN’S HEALTH, SEXUAL HEALTH, GERMANY

## Abstract

Unwanted pregnancies and abortions are experiences shared by many women. In light of the fact that some general framework conditions are currently changing in Germany, and that the Corona pandemic represents a particular challenge for the care of women with unwanted pregnancies, current data from the statistics on terminations of pregnancy of the Federal Statistical Office are outlined. Compared to Europe, Germany has a low proportion of induced abortions. In 2021, 94,596 abortions were reported. The number of abortions as well as the abortion rate and the abortion ratio have decreased since 2001. 95.8% of abortions took place according to the so-called counselling provision. In more than half of the abortions (52.1%) vacuum aspiration was used, in 11.4% curettage, 32.3% were medical abortions using mifepristone. There are large regional differences in the method used.

## Introduction

An unwanted pregnancy presents women with a decision situation, which can generally raise questions regarding their future life planning [1]. The decision to have an abortion is usually preceded by an intensive process of reflection. Many women experience unwanted pregnancies and abortions. Almost every sixth (16.8%) of the roughly 4,000 women between the ages of 20 and 44, who took part in the study ‘frauen leben 3’ by the Federal Centre for Health Education (BZgA) in 2012 [2], indicated that she had an unwanted pregnancy at least once. 42.9% of the women, who had become pregnant unwanted, had used contraception. Less than half (43.0%) of the unwanted pregnancies were terminated. Based on all women participating in the study, every twelfth woman (8.2%) had an abortion at least once in her life [2]. A ‘difficult partnership situation’ (34.0%) and ‘occupational and financial insecurity’ (20.3%) were specified thereby as most important reasons. The reasons ‘in training or studying’ (17.6%) and ‘young, immature’ (16.4%) were mostly given by younger women, ‘health-related concerns’ (19.7%) were mostly indicated by older women [2]. The proportion of women, who had an unwanted pregnancy, was significantly lower among women with high educational status than in the low education group. At the same time, unwanted pregnancies were terminated more frequently by women with higher education [2].

With roughly 4.5 abortions per 1,000 women, the proportion of terminations of pregnancy in Germany is low in European comparison [3]. According to the Statistical Office of the European Union (Eurostat), the highest abortion rates can be found in Georgia, Armenia and Bulgaria, but also the United Kingdom and Iceland have rates of more than ten abortions per 1,000 women of childbearing age [3]. The rate of the reported abortions is lowest in Poland, which has very restrictive legislation and virtually bans abortions completely – as a result, they are carried out illegally or women have to travel to other countries [4, 5]. For Germany, the Report on Women’s Health of the Robert Koch Institute (RKI) published in 2020 [6] describes, among others, that based on the data of the terminations of pregnancy statistics of the Federal Statistical Office [7], the number of reported terminations as well as the abortion rates (based on the number of women of childbearing age) and the abortion ratios (based on the number of live births) have decreased since 2001.

**Info box Exemption from punishment for abortion**

Under Section 218a (1) German Criminal Code (StGB) **(counselling provision)**, a termination of pregnancy will go unpunished if

► the pregnant woman requests the termination of the pregnancy,
► the pregnant woman has made use of pregnancy conflict counselling in accordance with Section 219 StGB, and obtained the counselling certificate there, and a three-day waiting period between counselling and procedure was adhered to,
► the termination of pregnancy is performed by a physician, and no more than twelve weeks have elapsed since conception. This corresponds to the fourteenth week of gestation, if not counting from the date of conception, but from the first day of the last menstrual period.

A termination of pregnancy is not unlawful if there are

► **medical grounds** (Section 218a (2) StGB): The termination of pregnancy is performed by a physician and, taking into account the present and future circumstances, is medically necessary to avert a danger to the life or the danger of grave impairment to the physical or mental health of the pregnant women; there is no time limit for terminations of pregnancy in case of medical grounds. There must be three full days between the medical diagnosis and the written documentation of medical grounds, unless the life of the pregnant women is in immediate danger. Before a physician issues a written determination of medical grounds, he has to counsel the pregnant women as to the medical and psychological aspects of an abortion and to inform her about the option of further psychosocial counselling and she has to confirm this in writing; physicians are obliged to provide contacts to appropriate counselling services upon request (Section 2a (2) and (3), SchKG).
► **grounds related to a crime** (Section 218a (3) StGB): The termination of pregnancy is performed by a physician. According to the physician’s knowledge, there are urgent reasons indicating that the pregnancy is a consequence of a rape or of sexual abuse; grounds related to crime always apply for all girls, who become pregnant before they reach the age of 14. Not more than twelve weeks must have passed since conception (14 weeks after the first day of the last menstrual period). The pregnant woman does not have to file a police report. There is no counselling obligation, but a right to counselling, if the pregnant woman wants this.

In both cases the termination of pregnancy must not be performed by the physician, who has issued the written determination stating the preconditions for an abortion on medical or other grounds.

Source: RKI, Report on Women’s Health, page 279 [6]; BZgA, www.familienplanung.de [13]

In Germany, abortion is generally illegal and thus punishable under Section 218 of the German Criminal Code (StGB) [8, 9]. There are three exceptions: The so-called counselling provision as well as the existence of medical grounds or grounds related to a crime (Info box). Section 219 StGB governs the counselling of pregnant women, whereby the content and the conducting of pregnancy conflict counselling are covered in the Act on Assistance to Avoid and Cope with Conflicts in Pregnancy (SchKG). The purpose of the pregnancy conflict counselling is to protect the unborn life, it is to be conducted with an open outcome, and is based on the responsibility of the woman. Pregnancy conflict counselling services have to be specially recognised by the State. More than 95% of the abortions take place under the counselling provision [6].

In February of 2019, a revised version of Section 219a StGB, which includes an advertising ban for abortions, entered into force. This allows physicians to indicate that they perform terminations. To enable physicians to publicly provide more detailed information about abortions without having to fear prosecution, the abolition of Section 219a was decided by the German Bundestag on 24 June 2022 [10, 11].

In addition, the Corona pandemic drew attention to the health care situation of women, who had an unwanted pregnancy, and the obstacles to find opportunities for counselling and for an abortion became increasingly apparent [12]. To counteract this, the option for pregnancy conflict counselling via digital media or by telephone was created [13]. At the end of 2020, a model project for the telemedical support of abortion at home was developed, which, as it turned out, was used especially by women in underserved regions [14]. In this project, the option of a medical abortion with mifepristone was used. This method is recommended by the WHO in addition to the method of vacuum aspiration [15]. So far, however, it is used comparatively rarely in Germany, in contrast to some other European countries [16].

The fact that women do not have a higher risk for developing mental health problems after a termination than women who have carried out a pregnancy, is now no longer in question [6]. The health and psychosocial care during and after an unplanned pregnancy as well as factors influencing the experience and coping with an unwanted pregnancy are currently being scientifically investigated in a large collaborative study (‘Experiences and life situations of people experiencing (un)planned pregnancies’, ELSA [17]). To improve care, terminations of pregnancy are to be included in the medical education and training [11], and the development of a medical guideline on safe abortion (evidence level S2k) was begun, which is to be completed in April of 2023 [18].

This fact sheet provides current data on abortions, also in light of the above-described current developments.

## Methodology

The Federal Statistical Office conducts the statistics on terminations of pregnancy quarterly. The legal basis is the Act on Assistance to Avoid and Cope with Conflicts in Pregnancy (SchKG) of July 27, 1992 (Federal Law Gazette I, p. 1398), last amended by Article 13a of the law of December 14, 2019 (Federal Law Gazette I, p. 2789). The focus of the statistics are the abortions carried out in Germany (in accordance with Section 16 SchKG); since 2010, the duration of the terminated pregnancies is reported in completed weeks. So-called reporting centres (Meldestellen), i.e. clinics and medical practices, where abortions are carried out, are obliged to provide the data. Overall, the statistics gives information on the magnitude, structure, and development of abortions in Germany as well as on selected living conditions of the women [19].

The number of abortions is shown below on the basis of current data for the year 2021, as well as the abortion rate (proportion according to the age of the women and in relation to 10,000 women of childbearing age) and the abortion ratio (in relation to 1,000 live births). The data on the number of women and live births are based on the statistics of natural movement of the population from the Federal Statistical Office, which provides information on changes in the number and structure of the population (e.g. with regard to the birth rate) [20]. Data on births are not yet available for 2021, so the information on abortions per 1,000 live births refers to the year 2020. The present article provides furthermore information on the development of abortions over time as well as on the reported abortions according to legal justification. It also informs about the reported abortions by duration of the terminated pregnancy, by marital status, and the number of previous live births, as well as by the location and type of the intervention.

## Results and discussion

In 2021, 94,596 abortions were carried out in Germany [19]. This corresponds to an abortion rate of 43.0 abortions per 10,000 women. Due to the fact that the number of women among a population can change, this information is especially relevant for comparisons of age groups or over time. To describe the relationship between terminated pregnancies and pregnancies that were carried to term, the abortion ratio (number of abortions per 1,000 live births) is used. For 2020, this amounts to 128.5 abortions per 1,000 live births. The number of the abortions and the abortion rate are very low among under 18-year-olds, while the abortion ratio is high. This means that girls under the age of 18 rarely become pregnant, but if they do, they are very likely to have an abortion. Among women aged 40 years and older, the number of abortions and the abortion rate per 10,000 women as well as the abortion ratio based on 1,000 live births are fairly low. This means that women from the age of 40 onwards rarely become pregnant but if they are pregnant, they are more likely to actually carry it to term (Table 1).

**Figure 1:**
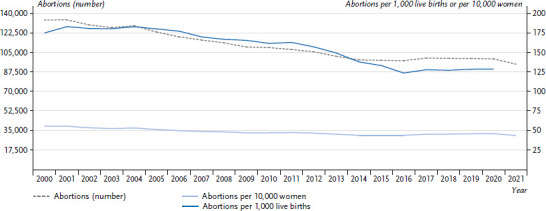
Abortions (number, per 10,000 women (aged 10 to 54) and per 1,000 live births) Source: Federal Statistical Office, statistics of terminations of pregnancy

Since the turn of the millennium, the number of reported abortions in Germany has been decreasing, from 134,964 in 2001 to 94,596 in 2021 (Figure 1) [19]. This corresponds to a decline of approximately 30%. Compared to the previous year, the number of abortions in 2021 declined by 5.4%. In 2020, which was likewise affected by the Corona pandemic, the number of abortions only declined by 0.9% [21].

The abortion rate of women of childbearing age (15 to 49 years) also decreased, from about 68 abortions per 10,000 women in 2001 to about 56 abortions per 10,000 women in 2021. At the same time, the abortion ratio referring to live births is also declining. This means that in the last 20 years, abortions have decreased more than births.

With 95.8%, the majority of abortions reported in 2021 was performed in accordance with the counselling provision. Abortions on medical grounds (4.1%) and on grounds related to a crime (0.05%) were considerably less frequent. The majority of abortions occurs early within the 12-week period: In 42.2% of the women, the gestational age was under seven weeks, in 33.6% it was seven to eight weeks, in 21.0% it was nine to eleven weeks. These proportions have not changed significantly since 2010.

With 58.2%, most of the women who had an abortion, were single; 38.0% were married, 3.8% were widowed or divorced. There were significant changes compared to 1996 with a higher proportion of married (52.3%) and a lower proportion of single women (40.6%). More than half of the women had already given birth to children: 21.7% had one child, 23.5% two, 13.9% three and more children, 40.9% of the women did not have children. The proportion of the women without children has increased slightly since 1996 (36.5%), the other proportions have decreased or remained the same.

Abortions are carried out almost exclusively on an outpatient basis: In 2021, 81.0% of the procedures took place in gynaecological practices or surgery centres, 15.7% took place on an outpatient basis in the hospital, 3.3% on an inpatient basis. In 1996, 13.6% of the procedures took place on an inpatient basis (52.1% in practices, 34.3% on an outpatient basis in the hospital) [7]. Regionalised data show differences between the women’s place of residence and the federal state, in which the abortion takes place. In more than one third (38.7%) of the women from Rhineland-Palatinate and in more than one sixth (18.6%) of the women from Lower Saxony, the abortion was carried out in a different federal state, mostly in Saarland or Bremen [19]. The number of the facilities performing abortions (so-called reporting centres) has been determined systematically by the Federal Statistical Office since the fourth quarter of 2018. In the fourth quarter of 2021, there were 1,092 reporting centres [22]. Their number has decreased sharply: In 1999, about 1,650 reporting centres, in 2003 about 2,050 reporting centres existed [23].

Vacuum aspiration is the method used predominantly for terminations of pregnancy, more than half (52.1%) of the abortions in 2021 were performed in this way. In 11.4% of the abortions a curettage is used, just under one third (32.3%) are medical abortions using mifepristone (other methods: 4.2%). Medical abortions with mifepristone have been reported in the statistics since 2000, then with a proportion of 3.1%, in 2019 – before the Corona pandemic – it was 25.0%. During this period, the proportion of vacuum aspirations has decreased significantly (2000: 82.6%, 2019: 56.9%), while the proportion of curettages has remained approximately the same with some fluctuations (2000: 11.2%, 2019: 14.1%). In addition, there are differences between the federal states: for example, the proportion of vacuum aspirations is highest in Rhineland-Palatinate (77.7%) and proportionately fewer medical abortions (12.5%), but also fewer curettages (8.8%) are carried out there than in the national average. In contrast, ahead of Berlin (51.6%), Schleswig-Holstein (52.9%) has the highest proportion of medical abortions, but the proportion of curettages (18.5%) is also significantly higher than the national average, while the proportion of vacuum aspirations (27.0%) lies below the national average. Hamburg has the lowest proportion of curettages (4.5%) [19]. It should be noted, however, that the abortion statistics only allows to report one method of abortion. In practice, however, it may happen that methods are combined (e.g. vacuum aspiration after medication), so that the corresponding proportions may be over- or underestimated.

When performed professionally, abortion has a very low risk of complications. In 2021, a total of 279 complications were reported, which corresponds to 0.29% of the procedures. Among these, secondary bleeding (30.8%) was the most common, with blood loss of more than 500ml in second place (27.6%) [19].

In summary, it can be stated that Germany is a country with a comparatively low and further decreasing rate of abortions. Whether the decrease in abortions in 2020 and the relatively strong decrease in 2021 are related to the Corona pandemic, cannot be determined at this point in time. The same applies to the significant increase in medical abortions. However, current figures also show that their proportion in Germany is still comparatively low at around one third; for example, medical abortions had a proportion of 79% in Switzerland [24] and 96% in Sweden (abortions before the ninth week) [25] in 2020, and 70% in France in 2019 [26]. Also, a relatively large percentage of curettages are still performed – with regional differences – although this method should no longer be used in the time up to 14 weeks of gestation according to WHO recommendations [15]. This could change through the teaching of skills and standards in medical education and training, as well as through the medical guideline on safe abortion, which is currently being worked on [18]. In Germany, the federal states are legally obliged to provide sufficient and professionally equipped facilities for the performance of abortions (Section 13(2) SchKG). However, the decreasing number of reporting centres and the proportion of abortions, which do not take place in the federal state, in which the women live, indicate that the supply and the accessibility of care have to be increased.

For women, the question of autonomy plays a central role in the discussion about unwanted pregnancies [1, 6]. According to the first Women’s Health Report of 2001, autonomy demands that ‘on the one hand, an improvement in the social framework conditions for living with children and, on the other hand, access to safe and women-friendly abortion options which cause as little physical and mental stress as possible, once a woman decides to have an abortion’ [1]. In addition to good sexuality education and good health information, low-threshold access so safe contraceptives can contribute to further reducing the number of unwanted pregnancies and therefore the number of abortions [6].

## Key statement

Compared to Europe, abortion rates in Germany are low; in 2021, 94,596 abortions were reported.

The number of abortions as well as the abortion rate and the abortion ratio have decreased since 2001.

95.8% of the abortions in 2021 took place according to the so-called counselling provision.

## Figures and Tables

**Table 1:** Number of abortions (based on all places of residence), abortion rates (based on women with permanent place of residence in Germany) and abortion ratios by age groups Source: Statistics of the terminations of pregnancy, statistics of the natural movement of population [7, 20]

Age group	Abortions		Abortions per 10,000 women^1^	Abortions per 1,000 live births
	Total	Women with place of residence in Germany		
	2021			2020
15–17 years	2,183	2,176	19.7	-
18–24 years	21,944	21,838	73.7	292.0
25–29 years	21,154	21,010	87.6	115.0
30–34 years	23,187	23,058	85.9	82.3
35–39 years	17,973	17,848	68.5	113.2
40–44 years	7,300	7,246	29.3	217.0
45–49 years	580	576	2.2	-
under 18 years	2,442	2,434	8.4	857.7
aged 45 years and older	596	592	1.0	313.0
aged 15–49 years altogether	94,321	93,752	55.8	-
**Total^2^**	**94,596**	**94,026**	**43.0**	**128.5**

^1^ Preliminary calculation on the basis of the population size in 2020

^2^ Women aged 10–54 years
